# Targeting Cell-Matrix Induced Chemoresistance With Regorafenib in a 3D Model of Osteosarcoma

**DOI:** 10.1002/jbm.a.37985

**Published:** 2025-09

**Authors:** Rameshwar R. Rao, Michelle S. Huang, Daiyao Zhang, Carla Huerta-López, Christopher Long, Giselle Aviles Rodriguez, Esther A. T. Mozipo, Sriya Sagi, Sarah C. Heilshorn

**Affiliations:** 1 Ben Towne Center for Childhood Cancer and Blood Disorders Research, Seattle Children's Research Institute, Seattle, Washington, USA; 2 Division of Pediatric Hematology, Oncology, Bone Marrow Transplant, and Cellular Therapies, Department of Pediatrics, University of Washington School of Medicine, Seattle, Washington, USA; 3 Department of Chemical Engineering, Stanford University, Stanford, California, USA; 4 Department of Materials Science and Engineering, Stanford University, Stanford, California, USA; 5 Department of Bioengineering, Stanford University, Stanford, California, USA

**Keywords:** 3D cancer models, osteosarcoma, regorafenib, tyrosine kinase inhibitors

## Abstract

Over the past four decades, there has been little advancement in treatment strategies for osteosarcoma (OS), the predominant primary bone tumor in the pediatric patient population. Current therapy involves multiple rounds of chemotherapy and surgical resection, which are associated with significant morbidity and suboptimal survival rates. A key challenge in developing new treatments is the difficulty in replicating the OS tumor microenvironment, particularly cell interactions with the extracellular matrix (ECM). This study uses an in vitro model of OS to investigate the cell response to collagen (COL) type I, the primary component of the OS ECM. After 7 days of culture within three-dimensional COL hydrogels, OS cells displayed a more elongated cellular morphology and reduced sensitivity to the standard chemotherapy used for OS treatment compared to cells grown on two-dimensional substrates. To test whether this model could be used to study treatment strategies used for high-risk OS patients, we applied a metronomic regimen combining regorafenib, a multi-tyrosine kinase inhibitor, with front-line chemotherapy to overcome cell-matrix induced chemoresistance. We identified overexpression of the ATP-binding cassette transporter ABCG2, a drug efflux pump, as a potential mechanism of resistance in 3D culture. Regorafenib's inhibitory effect on ABCG2 suggests a mechanistic basis for its ability to restore chemosensitivity in 3D culture. Altogether, these findings highlight the importance of cell–matrix interactions in in vitro OS models, provide valuable insights into a matrix-induced mechanism of OS chemoresistance, and suggest an approach to its treatment.

## 1 ∣ Introduction

Osteosarcoma (OS) is the primary bone tumor diagnosed in pediatric patients and young adults, with 400 new cases annually in the US [1]. The therapy for OS involves intensive chemotherapy consisting of multiple cycles of methotrexate (MTX), doxorubicin (DOX), and cisplatin (CIS) [2]. Despite this aggressive therapy, localized non-metastatic OS has a 5-year survival rate of 60%, and metastatic, refractory, or recurrent disease has a dismal 20% survival rate [3]. While many drug and cellular-based therapies are being explored to treat metastatic and relapsed OS patients [4], limited progress has been achieved in improving survival outcomes for patients with OS.

The OS tumor microenvironment (TME) plays a crucial role in tumor development and progression [5]. The malignant bone microenvironment is a complex and dynamic system, comprising various cell types such as osteoblasts, bone marrow stromal cells, hematopoietic cells, endothelial cells, immune cells, and malignant cells [6]. This heterogeneous cell population within the TME facilitates cell–cell interactions that modulate multiple cell signaling pathways, driving OS growth and metastasis [7]. Additionally, immune cells within the OS TME regulate anti-tumor activity through a balance of proinflammatory and immunosuppressant cytokines [8]. While this has led to multiple investigations in the use of immunotherapy with immune checkpoint inhibitors and adoptive cell therapies, these approaches have yet to establish a durable response for relapsed patients in clinical trials [9, 10].

In addition to the cellular components, the TME includes an altered extracellular matrix (ECM), which plays a pivotal role in tumor development and progression. The ECM forms a three-dimensional (3D) acellular physical environment that not only provides structural support but also regulates cellular functions such as signaling, migration, proliferation, adhesion, and differentiation through both biochemical and biomechanical signaling [11]. OS generates a robust ECM composed of proteinaceous components like collagen type 1 (COL), laminin, and fibronectin, as well as proteoglycans such as hyaluronan [12]. Interactions between the diverse cell types within the OS TME and constituents of the ECM drive signaling pathways responsible for ECM remodeling and malignant cell migration, metastasis, and chemoresistance [13].

One major challenge in developing effective new treatments for OS is the lack of representative in vitro tumor models to study TME interactions. While patient-derived xenograft (PDX) models encompass all components of the TME [14], these systems have significant limitations including high cost, lengthy development times (ranging from months to a year) and low tumor engraftment rate [15]. On the other hand, traditional cell culture methods that involve growing cells on top of tissue culture plastic (TCP) in a two-dimensional (2D) monolayer are cost-effective and time-efficient, but lack a 3D ECM [16].

3D cell culture platforms, where cells are cultured within a 3D support matrix that mimics the in vivo microenvironment, bridge the gap between 2D TCP culture and PDX models [17]. Unlike PDX models, tissue-engineered 3D models of cancer allow for systematic tuning of the TME by selecting specific cellular or matrix components to study [18]. While 3D in vitro models do not encompass all components of the TME, this reductionist model can be advantageous for understanding the cause-and-effect relationship behind specific cell–matrix interactions. Emerging evidence suggests that 3D in vitro models of cell–matrix interactions can produce differential cell responses that more accurately model tissue development, homeostasis, and pathogenesis compared to traditional 2D in vitro models [19].

In the context of OS, 3D cell culture systems have recently been developed, both within natural and synthetic biomaterials [20]. These in vitro models can provide a valuable platform for understanding the mechanisms driving OS progression and treatment resistance. For example, 3D models of OS are being employed to understand various stages of OS development and the impact of oxygen tension on OS cell response [21, 22]. Interestingly, several of these models have reported significant differences in cell growth and phenotype compared to 2D monolayer cultures [23-26]. In particular, some 3D models have suggested that 3D OS cell lines exhibit decreased response to chemotherapy that may be relevant to clinically observed chemoresistance [23, 27, 28]. The molecular mechanisms underlying this decreased chemosensitivity in 3D cultures remain largely unknown.

In other cancer types, there continues to be accumulating evidence within the cancer tissue engineering field that cell–matrix interactions yield an increase in ATP-binding cassette (ABC) transporter expression [29, 30]. The ABC transporters are transmembrane proteins responsible for transporting substrates across the lipid bilayer of the cell membrane. These transporters play a significant role in drug efflux, which affects the intracellular concentrations of certain drugs. The expression of ABC transporters can influence tumor phenotype and contribute to chemoresistance. In OS, three key transporters, ABCB1 (P-glycoprotein, P-GP/multi-drug resistance protein, MDR1), ABCC1 (multi-drug resistance associated protein 1, MRP1), and ABCG2 (breast cancer resistance protein, BCRP) are known to promote chemoresistance by directing chemotherapy efflux from malignant cells [31]. Retrospective OS trials have shown that overexpression of ABC transporters can predict poor patient outcomes, and a recent prospective clinical trial demonstrated benefit in intensifying therapy in ABCB1+ patients [32].

**FIGURE 1 ∣:**
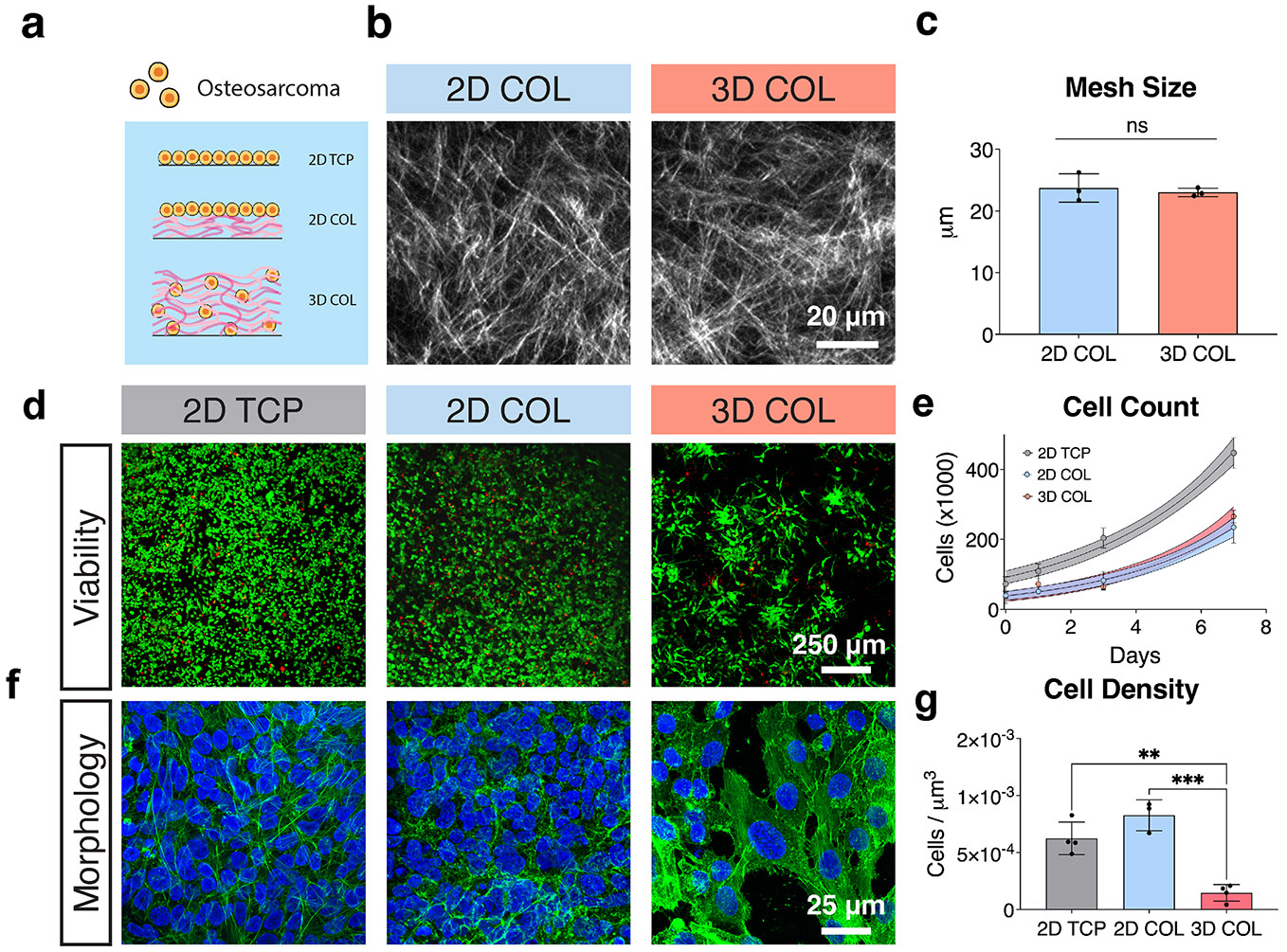
OS cell behavior across 2D and 3D culture. (a) Schematic of OS cell culture conditions: 2D TCP, 2D COL, and 3D COL. (b) Representative SHG images of acellular 2D COL and 3D COL. Scale bar = 20 μm. (c) Quantification of mesh size revealed no significant differences between 2D COL and 3D COL matrices (*n* = 3 independent technical replicates, mean ± SD, unpaired two-tailed *t*-test, ns = no significant differences). (d) Live/Dead staining of OS cells cultured in 2D TCP, 2D COL, and 3D COL conditions on day 7, showing high cell viability across all conditions (green—Live cells, red—Dead cells). Scale bar = 250 μm. (e) Cells proliferated in all conditions, with similar growth velocities in 2D COL and 3D COL matrices (*n* = 4 independent technical replicates, nonlinear growth curve with shading representing 95% confidence interval). (f) At day 7, F-Actin staining (green) and nuclear visualization (blue) revealed confluent cell monolayers in 2D TCP and 2D COL, and elongated cells with stretched F-Actin fibers within 3D COL matrices. Scale bar = 25 μm. (g) Quantification of cell density at day 7 based on confocal microscopy *z*-stacks indicated no significant difference between 2D TCP and 2D COL, but a marked decrease in cell density for 3D COL conditions (*n* = 4 independent technical replicates, mean ± SD, ordinary one-way ANOVA with Tukey's post hoc analysis, **p < 0.01, ***p < 0.001). Abbreviations: OS = osteosarcoma; 2D = two-dimensional; 3D = three-dimensional; TCP = tissue culture plastic; COL = collagen; SHG = second harmonic generation; SD = standard deviation; ANOVA = analysis of variance.

Motivated by these prior studies, the goal of this work was to evaluate the chemosensitivity of OS cells to frontline therapy in a traditional 2D culture model, a modified 2D model that enables cell–matrix interactions along a planar interface, and a 3D model that enables cell–matrix interactions across the entire cell surface (Figure 1a). This unique experimental design allowed us to explicitly test the hypothesis that the dimensionality of cell–matrix interactions alters chemosensitivity. Furthermore, our 3D COL model tested a clinically relevant treatment regimen incorporating a multi-tyrosine kinase inhibitor to rescue chemosensitivity and identified overexpression of ABCG2, a drug efflux pump known to be inhibited by regorafenib, as one potential mechanism of chemoresistance. Overall, this work demonstrates that 3D COL culture models can activate mechanistic pathways of known clinical relevance and can provide a potential pathway for developing more effective therapeutic strategies that improve drug efficacy.

## 2 ∣ Materials and Methods

### 2.1 ∣ Collagen Hydrogel Synthesis

Stock COL for coatings and hydrogels was prepared by dissolving lyophilized bovine type 1 COL (Fisher Scientific) in 0.02 N acetic acid at 4°C for 48 h to produce an 8.0 mg/mL stock COL solution. To fabricate COL hydrogels, the stock solution was mixed with 10% Minimum Essential Medium α (Invitrogen), 20% 10×-concentrated MEM α, 10% fetal bovine serum (FBS, Invitrogen), and 10% 0.1 N sodium hydroxide to achieve a final COL concentration of 4.0 mg/mL. For 2D COL coatings, 100 μL of this solution was added to a 48-well plate (approximate gel thickness of 1330 μm), while 200 μL was used to form a 3D COL disc (approximate gel thickness of 2670 μm). The constructs were then incubated at 37°C for 30 min to ensure homogeneous gelation. For cell experiments, 250 μL of media was added into each well after the 30-min incubation period.

### 2.2 ∣ Second Harmonic Generation Imaging

The structural parameters of the fibrillar collagen networks were visualized using second harmonic generation (SHG) imaging. An inverted microscope (Nikon, Ti2-E equipped with a C2 confocal scanning head and a Nikon CFI Apochromat TIRF 100XC oil immersion objective) was used for this purpose. The SHG signal was generated by stimulating the samples with an external laser beam generated by a picosecond-pulsed laser system (APE America Inc., picoEmerald S with 2 ps pulse length, 80 MHz repetition rate, and 10 cm^−1^ bandwidth) consisting of a 1031 nm mode-locked ytterbium fiber laser and an optical parametric oscillator (OPO) tunable between 700 and 960 nm. The OPO wavelength was set to 797 nm, and the backscattered SHG signal (at a wavelength of 398.5 nm) was separated using a set of optical filters (BrightLine 400/12 bandpass, BrightLine 390/18 bandpass, Thorlabs FESH0500 shortpass) and detected pixel-by-pixel with a photomultiplier tube (Hamamatsu, R6357). The excitation power at the sample was 25 mW.

For each gel, five image *z*-stacks were acquired as technical replicates. Each stack comprised 21 slices spaced 1 μm apart for an effective imaging depth of 20 μm. Each image was acquired at a resolution of 1024 × 1024 pixels (77.82 μm × 77.82 μm) with a dwell time of 10.8 μs/pixel.

All SHG image *z*-stacks were analyzed using a combination of the Fiji distribution of ImageJ and a modified version of a MATLAB script developed by Rossen et al. [33] The *z*-stacks were processed using an east shadows filter and 3D Gaussian blur filtering (*σ* = 1). The COL fibrils were thresholded from the background signal using Otsu’s method [34]. The resultant binary mask was then multiplied by the original processed image to effectively isolate the fibrils from the background. The contour length, persistence length, network mesh size, and number of fibrils were then calculated by the MATLAB script as described in Rossen et al. [33], with a length prioritization array of [30 15 10], a cone angle of 30°, and a stop parameter of 0.4. To calculate fibril thickness, the “Local Thickness” plugin in Fiji was used.

### 2.3 ∣ Cell Culture

143B human OS cells were obtained from the American Type Culture Collection and cultured in Minimum Essential Medium α supplemented with 10% FBS and 1% penicillin–streptomycin (Invitrogen). Cells were cultured at 37°C in a 5% CO_2_ atmosphere, with media changes every other day. Cells were used between passages 4 and 9.

Cells were dissociated from tissue culture flasks with TrypLE Express, and samples were generated for the three conditions at the same time and passage for each experiment. Cells were seeded on 2D TCP and 2D COL coatings at a 2D cell seeding density of 100,000 cells per well (91,000 cells/cm^2^). For 3D COL hydrogels, cells were encapsulated at a 3D volumetric cell density of 500,000 cells/cm^3^. 3D COL hydrogels were formed using a total volume of 200 μL of gel mix, resulting in 100,000 cells per hydrogel (0.2 mL of a 500,000 cells/mL solution). Thus, the total number of seeded cells per well for the 2D TCP, 2D COL, and 3D COL conditions was kept constant at 100,000 cells per well. Media were refreshed every other day throughout the culture period.

### 2.4 ∣ Cell Viability Assay

Cell viability was determined using a Live/Dead kit (Invitrogen) and performed at 37°C. Samples were washed 3 times in phosphate buffered saline (PBS, Invitrogen) for 5 min per wash and then incubated with 4.0 μM calcein-AM and 4.0 μM ethidium homodimer-1 in PBS for 30 min. Samples were washed again in PBS and then imaged on a confocal microscope (Leica STELLARIS 5) at 10× magnification. Images were captured with *z*-stacks with 10 slices per image through a height of 150–200 μm within the hydrogel. Viability was quantified using Fiji software (NIH, version 2.14) by applying intensity thresholds to differentiate and count live cells (green) from dead cells (red). Three images were taken per sample, and four samples were averaged for each condition.

### 2.5 ∣ Cell Growth Analysis

Cell proliferation was quantified using an automatic cell counter (Countess 3, Invitrogen) at days 0 (immediately after gelation), 1, 3, and 7 of culture. Cells grown on 2D TCP were treated with TrypLE (Invitrogen) for 5 min at 37°C to detach cells and then quantified. For 2D COL and 3D COL samples, COL was degraded by incubating the samples at 37°C with 1 mg/mL collagenase derived from *Clostridium histolyticum* (Fisher Scientific) for 2 h, after which cells were counted. Each sample was quantified using two independent counts to ensure accuracy. This concentration of collagenase was chosen as previous work has demonstrated no significant impact on cell viability after treatment with 1 mg/mL collagenase [35].

### 2.6 ∣ Cell Morphology and Cell Density

Cell morphology was assessed via immunocytochemistry on day 7 of culture. Samples were first washed with PBS for 10 min, followed by fixation with pre-warmed 4% paraformaldehyde at 37°C for 30 min. Samples were then washed three times with PBS and permeabilized with 0.25% v/v Triton X-100 in PBS. Nuclei were stained with DAPI (1:1000 dilution), and F-actin was labeled with Alexa-Fluor 647 Phalloidin (1:400 dilution) in a solution containing PBS, 2.5% w/v BSA, 2.5% v/v goat serum, and 0.5% v/v Triton X-100. Samples were incubated overnight at 4°C on a shaker. Samples were washed three times in PBS with Triton X-100 for 30 min per wash and then imaged using a confocal microscope (Leica STELLARIS 5) at 63× magnification. Images were captured with *z*-stacks with 10 slices per image through a height of 5 to 20 μm within the hydrogel. Cell density was calculated by counting the nuclei per image and dividing by the *X*, *Y*, and *Z* dimensions of each image.

### 2.7 ∣ Quantitative Real-Time Polymerase Chain Reaction

At day 0 (immediately after gelation) or day 7 of culture, samples were processed for RNA extraction. Cells cultured on 2D TCP were treated with TrypLE for 5 min at 37°C, while cells in 2D COL and 3D COL conditions were degraded using 1 mg/mL collagenase for 2 h at 37°C. Cells were then centrifuged at 1000 RPM for 5 min, and the supernatant was aspirated. The cell pellet was homogenized in 250 μL of Trizol reagent (Invitrogen) using sonication to optimize RNA extraction. RNA was isolated using Phase Lock Gels (Quantabio) in conjunction with chloroform, and RNA concentration and purity were quantified using a NanoDrop spectrophotometer (Invitrogen). A fixed amount of RNA (0.1–1 μg) from each sample was reverse-transcribed into cDNA using the High-Capacity cDNA Reverse Transcription Kit (Applied Biosystems). The cDNA was then amplified using Fast SYBR Green Master Mix (Applied Biosystems) with specific forward and reverse primers (Integrated DNA Technologies, Table S1). Quantitative real-time polymerase chain reaction was performed using the StepOnePlus Real Time PCR System (Applied Biosystems) and cycle threshold (Ct) values were calculated. Relative gene expression was reported by the ΔΔCt method.

### 2.8 ∣ Alkaline Phosphatase Activity and DNA Quantification

Intracellular alkaline phosphatase (ALP) activity was quantified using a fluorometric Alkaline Phosphatase Assay Kit (Abcam). For cells cultured on 2D TCP, TrypLE was applied for 5 min at 37°C to detach cells. In 2D COL and 3D COL conditions, COL was degraded with 1 mg/mL collagenase derived from *Clostridium histolyticum* for 2 h at 37°C. Samples were then centrifuged at 1000 RPM for 5 min, and the supernatant was discarded. The cell pellets were lysed on ice for 2 h in a lysis buffer consisting of 10 mM Tris buffer (Sigma) at pH 7.4, 0.2% v/v IGEPAL (Sigma), 2 mM phenylmethanesulfonylfluoride (Sigma), and 0.02% v/v Triton X-100. A 50 μL aliquot of the lysate was used in the assay to measure ALP activity on day 1. ALP activity was normalized to DNA content determined through the PicoGreen assay (Life Technologies).

### 2.9 ∣ Response to Chemotherapy

Cells were cultured on 2D TCP, 2D COL, and within 3D COL matrices for 7 days before being treated with 12 concentrations of either MTX, DOX, or CIS for 24 h. Each chemotherapy drug was dissolved in dimethyl sulfoxide and diluted within cell culture media. A final volume of 250 μL of media with chemotherapy was added to each sample in the three culture conditions. Cellular metabolic activity, used as an indicator of chemotherapy response, was determined through the alamarBlue Cell Viability Assay (Invitrogen). The alamarBlue reagent was diluted 1:10 in cell culture media and incubated with samples for 4 h. Then 100 μL of media was then read on a fluorescent plate reader at an excitation wavelength of 560 nm and an emission wavelength of 590 nm. Fluorescence values were normalized to the 0 μM drug condition to determine relative cellular metabolic activity.

### 2.10 ∣ Fluorescence Recovery After Photobleaching

Fluorescence recovery after photobleaching was conducted to evaluate diffusivity through the hydrated COL matrix. COL hydrogels were cast in a black, half-area 96 well-plate, and fluorescein isothiocyanate-dextran probes (Sigma) of various molecular weights (10, 20, 40, 75, 150, and 500 kDa) were dissolved in PBS at a concentration of 4 mg/mL and incubated with the hydrogels overnight to allow for probe diffusion. The hydrogels were then imaged within the hydrogel at a *z*-distance of 100 μm above the bottom of the gel and photobleached using a Leica STERLLARIS 5 confocal microscope. A 100 μm × 100 μm region was bleached for 1 min with a 488 nm laser set at 100% intensity. Images were then captured over a 4-min recovery period with the laser intensity reduced to 10%. The diffusivity of the probes through the COL matrix was calculated using an open-source MATLAB code “frap_analysis,” which employs the Hankel transform method.

### 2.11 ∣ Doxorubicin Nuclear Intercalation

Cells were cultured for 7 days on 2D TCP, 2D COL, and within 3D COL before being treated with 25 μM DOX for 24 h. Samples were first washed with PBS for 10 min, followed by fixation with pre-warmed 4% paraformaldehyde at 37°C for 30 min. Samples were then washed three times with PBS and permeabilized with 0.25% v/v Triton X-100 in PBS. Samples were then incubated with DAPI and Alexa-Fluor 647 Phalloidin as described above. Confocal microscopy (Leica STELLARIS 5) at 63× magnification was used to image the samples, with DOX localization within the nuclei visualized using an excitation wavelength of 470 nm and an emission wavelength of 560 nm.

### 2.12 ∣ Western Blot

Protein expression of ABCG2 was determined at day 7 of culture. Samples were collected in either TrypLE (2D TCP) or in 1 mg/mL collagenase (2D COL and 3D COL). Cells were concentrated by centrifugation at 1000 RPM for 5 min, and supernatant was removed. Samples were resuspended in 100 μL of radioimmunoprecipitation assay lysis buffer solution supplemented with 1 mM phenylmethanesulfonylfluoride and protease inhibitor tablets (Roche), incubated on ice for 20 min, and frozen at −80°C until use. After thawing on ice, 20 μL of lysate was combined with 5 μL of 5× Laemmli buffer (50% v/v) glycerol, 10 wt % sodium dodecyl sulfate, 0.05 wt % bromophenol blue, 300 mM tris (pH 6.8), and fresh 500 mM dithiothreitol). Samples were incubated at 96°C for 10 min to denature protein. Proteins were separated by SDS-PAGE and transferred onto polyvinylidene fluoride membranes (0.45 μm, Invitrogen) via wet transfer. Membranes were blocked for 1 h in blocking solution: 5 wt % milk in tris-buffered saline (20× stock: 3 M NaCl and 750 mM tris hydrochloride (pH 7.2) supplemented with 0.25% (v/v) Tween-20 (TBST). Samples were incubated with primary antibodies (Table S2) overnight at 4°C. The next day, the membranes were washed three times with TBST and treated with horseradish peroxidase-conjugated secondary antibodies (1:10,000; Jackson ImmunoResearch) for 1 h at room temperature. The membranes were washed three times with TBST, developed using either the SuperSignal West Pico or Femto Chemiluminescent Substrate (Thermo Fisher Scientific), and imaged using a ChemiDoc MP gel imaging system (Bio-Rad). Densitometry analysis was performed using ImageJ to quantify the blots.

### 2.13 ∣ Rheology

Rheological measurements were conducted on acellular COL gels using an AR-G2 rheometer (TA Instruments). The gel mixture was initially loaded on the rheometer at 4°C. A temperature ramp was applied to gradually raise the temperature to 37°C. A time sweep was then performed for 30 min at 37°C to ensure full gelation. Subsequently, a frequency sweep was carried out over a range of 0.1–10 rad/s at 1% strain. The storage modulus was recorded at a frequency of 1 rad/s during the frequency sweep to assess the mechanical properties of the gel.

### 2.14 ∣ Statistical Analysis and Reproducibility

Statistical analyses were conducted using GraphPad Prism v. 10.2.3. Quantitative data were analyzed using an unpaired two-tailed *t*-test, an ordinary one-way Analysis of Variance (ANOVA), followed by Tukey's post hoc analysis, or mixed-effects two-way ANOVA test, followed by Tukey's post hoc analysis. Statistical significance was denoted as follows: not significant (ns, *p* > 0.05), *p < 0.05, **p < 0.01, ***p < 0.001, and ****p < 0.0001.

## 3 ∣ Results

### 3.1 ∣ Collagen Material Properties and Dimensionality

The predominant ECM component of the unmineralized OS tumor microenvironment (malignant osteoid) is COL type I. To evaluate if in vitro OS cultures respond to the dimensionality of collagen substrates, we prepared in vitro tissue models with both 2D and 3D configurations, using TCP as a control. COL concentration was held constant at a final concentration of 4 mg/mL in both the 2D and 3D configurations to standardize integrin ligand density and mechanical stiffness, while only the dimensionality was varied. Rheological characterization revealed COL hydrogels formed within 3 min of incubation at 37°C, as indicated by the crossover of storage and loss moduli during the temperature sweep (Figure S1). The hydrogels exhibited an average shear storage modulus of ~800 Pa at a frequency of 1 rad/s (Figure S1). Assuming a Poisson's ratio (ν) of 0.5 for incompressible materials, COL gels prepared in this manner have an elastic modulus (i.e., stiffness) of ~2.4 kPa.

We then characterized the microarchitecture of the COL hydrogels in both 2D and 3D through SHG microscopy (Figure 1b). This nonlinear optical imaging technique allows for visualization of COL fibril/fiber structure without the use of exogenous labels [36]. As expected, both conditions displayed disorganized COL fibers characteristic of the native malignant ECM [37] with similar mesh size (Figure 1c) independent of gel dimensionality. Local mesh size remained similar in both 2D COL and 3D COL after cell encapsulation and was not significantly different compared to acellular hydrogels in both COL culture conditions (Figure S2). While the fibrillar organization of the COL was preserved in both systems, cells cultured in the 3D model were encapsulated within the COL matrix, allowing integrin binding in all planes of contact, while cells in the 2D configuration only interacted with the COL at the surface of the matrix.

### 3.2 ∣ Cell Viability, Cell Metabolic Activity, Cell Proliferation, Cell Morphology, and Cell Density

143B cells, a highly metastatic, *k-ras* activated OS cell line derived from a female adolescent patient, were used within this study [38]. The initial number of seeded cells was kept constant across all three conditions (2D TCP, 2D COL, and 3D COL). Cell viability was assessed on day 7 of culture through a Live/Dead Viability Stain. On day 7 of culture, OS cells demonstrated high viability (> 95%), with no significant differences in viability between the three culture conditions (Figures 1d and S3). In the 3D COL condition, cells were homogenously distributed at day 0, while multicellular units were observed at day 3 and 7 of culture throughout the depth of the hydrogel (Figures S4 and S5), suggesting that cell proliferation was occurring. To estimate cell proliferation rates, we analyzed the number of cells across the three conditions over time (Figure 1e). Proliferation was faster on 2D TCP, consistent with observations from others that more rigid substrates can increase cell proliferation rates [39]. We observed similar growth curves for 2D and 3D COL cultures, suggesting similar proliferation rates for these two conditions. At day 7, OS cells had formed a confluent monolayer in both 2D culture conditions with a cobblestone morphology on top of the culture substrate (Figure 1f). In contrast, in the 3D COL environment where the cells were surrounded by a COL matrix, the cells adopted a more elongated morphology, with stretched F-actin fibers, and appeared to proliferate to form multicellular units.

We hypothesized that since cells in the 3D COL model can expand throughout the height of the gel, they would have more physical space to grow and hence be at a lower cell density. To compare cell density across the three culture conditions, we first used confocal microscopy *z*-stacks spanning the entire cell monolayer in the 2D culture conditions (~10 μm) to count the number of cell nuclei. We then used similar ~10-μm *z*-stacks in the 3D COL condition to count the number of cell nuclei within a volume of hydrogel. As expected, cell density was significantly lower in 3D COL compared to 2D COL, while cell density in the two 2D conditions was statistically similar (Figure 1g).

### 3.3 ∣ Upregulation of Alkaline Phosphatase Activity and Activation of the Phosphatidylinositol-3 Kinase /AKT Pathway

**FIGURE 2 ∣:**
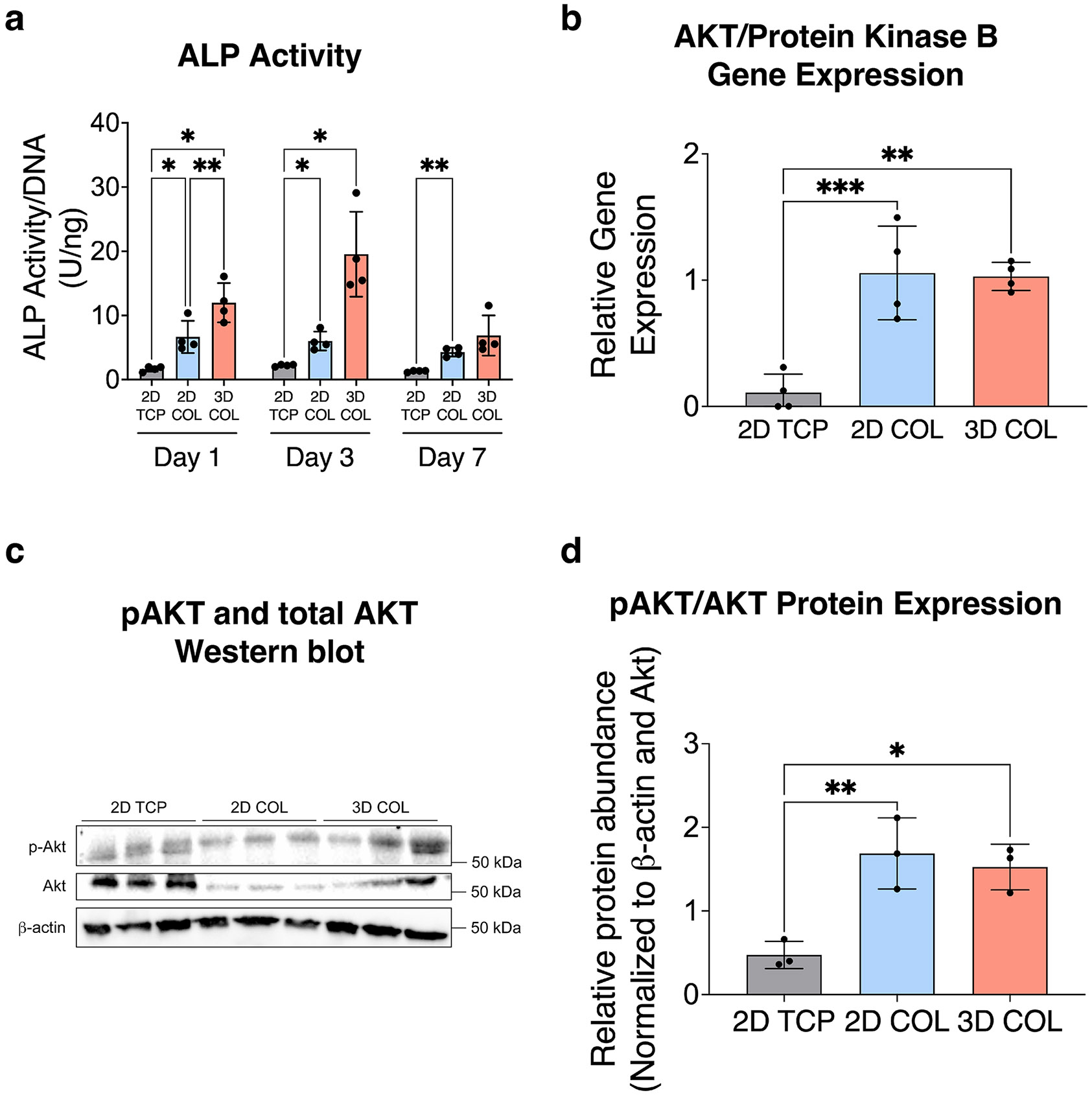
Increased expression of ALP and activation of the PI3K/AKT pathway in OS cultures within 3D COL matrices. (a) ALP activity, a biomarker of OS, was significantly higher in 2D COL and 3D COL cultures compared to 2D TCP at days 1 and 3 of culture compared to 2D TCP (*n* = 4 independent technical replicates, mean ± SD, ordinary one-way ANOVA with Tukey's post hoc analysis, *p < 0.05, **p < 0.01). (b) AKT/Protein Kinase B gene expression was significantly increased in 2D COL and 3D COL cultures compared to 2D TCP (*n* = 4 independent technical replicates, mean ± SD, ordinary one-way ANOVA with Tukey's post hoc analysis, **p < 0.01, ***p < 0.001). (c and d) Western blot and protein expression of activated AKT (pAKT) and total AKT demonstrated increased activated AKT normalized to total AKT in 2D COL and 3D COL cultures compared to 2D TCP (*n* = 3 independent technical replicates, mean ± SD, ordinary one-way ANOVA with Tukey's post hoc analysis, * *p* < 0.05, **p < 0.01). Abbreviations: ALP = alkaline phosphatase; PI3K = phosphatidylinositol-3 kinase; OS = osteosarcoma; 3D = three-dimensional; COL = collagen; 2D = two-dimensional; TCP = tissue culture plastic; SD = standard deviation; ANOVA = analysis of variance.

We next quantified intracellular alkaline phosphatase (ALP) activity across the three conditions at days 1, 3, and 7 of culture (Figure 2a). ALP is essential for osteoblast differentiation and ECM mineralization. As OS produces osteoid tissue and immature bone, serum ALP is increased in OS patients at diagnosis [40]. Quantification of ALP activity normalized to DNA content showed ALP activity was upregulated in both 2D COL and 3D COL cultures at days 1 and 3, but was also significantly increased in 3D COL compared to 2D COL at day 1 of culture.

We hypothesized that because COL is present in both the 2D COL and 3D COL models, both of these in vitro systems would facilitate engagement through integrin subunit β1, a cell surface receptor involved in collagen binding, and initiate downstream cellular signaling indicative of cell–ECM interactions [41]. Among the signaling pathways involved in OS pathogenesis, the phosphatidylinositol-3 kinase (PI3K)/AKT pathway is particularly prominent and downstream of integrin subunit β1 signaling [42]. This pathway regulates malignant cell proliferation, inhibits apoptosis, and promotes pulmonary metastasis [42]. To confirm the bioactivity of COL in our model, we performed gene and protein expression analysis of AKT, the downstream effector of the PI3K/AKT pathway. Gene expression of *AKT* was significantly elevated at day 7 of culture in the 2D COL and 3D COL culture conditions compared to 2D TCP (Figure 2b). We further confirmed protein expression of phosphorylated-AKT (pAKT, i.e., activated AKT) and total AKT through Western blot in the three culture conditions to determine the normalized activated AKT levels (Figure 2c,d). Similar to gene expression results, the activated, phosphorylated-AKT levels were increased in both the 2D COL and 3D COL culture conditions compared to 2D TCP. These findings suggest that the COL matrix can effectively induce cell-receptor engagement and downstream signaling of the PI3K/AKT pathway in both 2D COL and 3D COL cultures.

### 3.4 ∣ Response to Front-Line Chemotherapy

**FIGURE 3 ∣:**
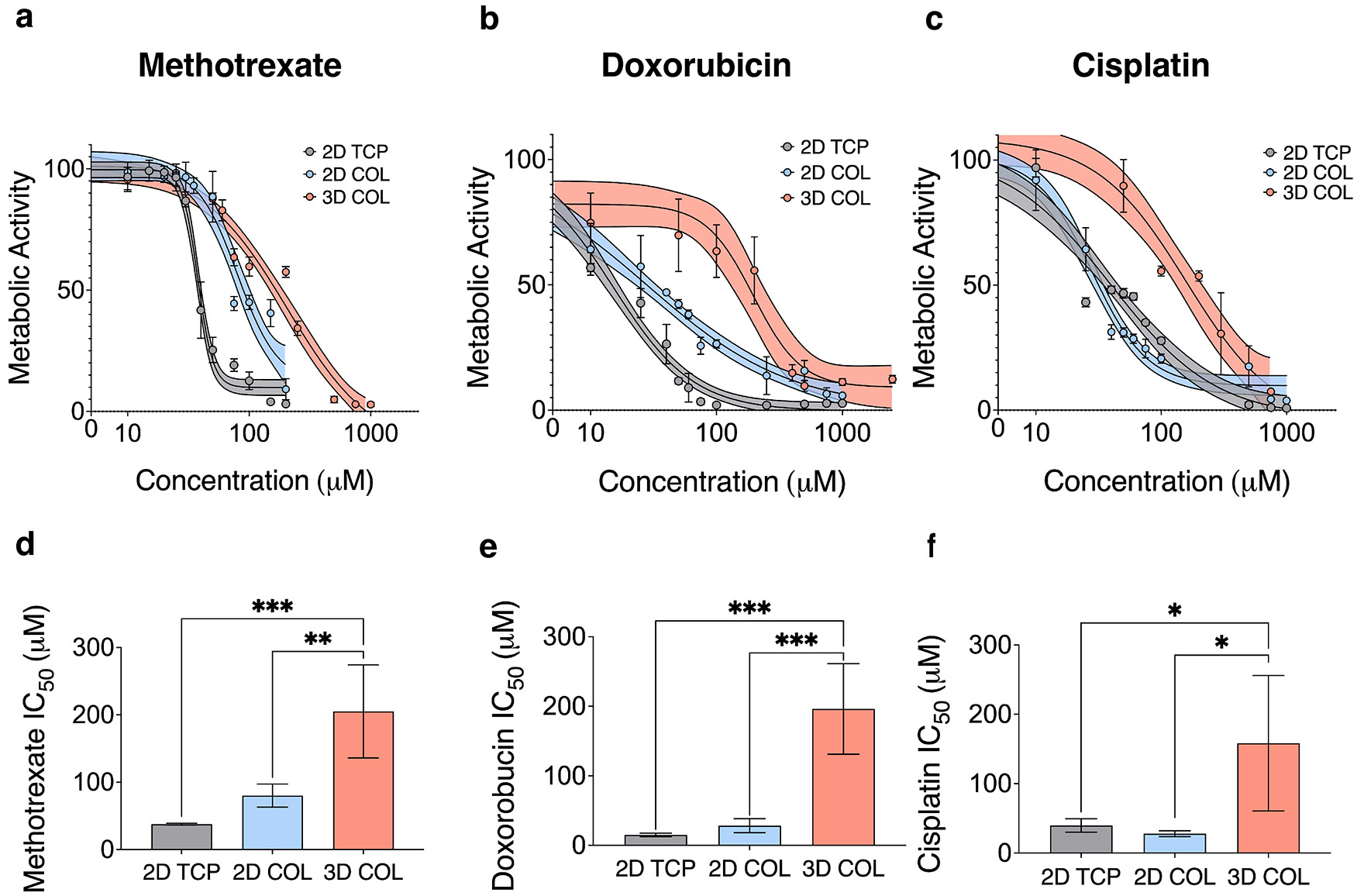
Decreased chemosensitivity in OS cultures within 3D COL matrices. (a–c) Dose–response curves after 24 h of treatment with MTX, DOX, and CIS revealed an increase in the half maximal inhibitor concentration (IC_50_). (d–f) in response to each drug for the 3D COL culture condition, denoting decreased chemosensitivity compared to 2D TCP and 2D COL (*n* = 4 independent technical replicates, mean ± SD, (a–c)—center solid line is nonlinear least squares regression of data, shaded region represents 95% confidence bands of the nonlinear fit, (d–f)—IC_50_ values calculated from nonlinear fit of dose–response curves, ordinary one-way ANOVA with Tukey's post hoc analysis, *p < 0.05, **p < 0.01, *** *p* < 0.001). Abbreviations: OS = osteosarcoma; 3D = three-dimensional; MTX = methotrexate; DOX = doxorubicin; CIS = cisplatin; 2D = two-dimensional; TCP = tissue culture plastic; SD = standard deviation; ANOVA = analysis of variance.

The standard treatment regimen for both localized and metastatic OS remains chemotherapy with methotrexate (MTX), adriamycin (doxorubicin, DOX), and cisplatin (CIS), often referred to as MAP therapy when applied as sequential cycles clinically [43]. In this study, OS cells were cultured on 2D TCP, 2D COL, and within 3D COL environments for 7 days, allowing for cell attachment, growth, and confluency in the 2D conditions or the formation of multicellular aggregates in the 3D condition. After this period, the cells were exposed to 12 different concentrations of each chemotherapy agent for 24 h, and metabolic activity was measured as an indicator of the OS cellular response to treatment. Dose–response curves revealed a higher half-maximal inhibitory concentration (IC_50_) in 3D COL cultures for each drug tested: MTX (Figure 3a,d), DOX (Figure 3b,e), and CIS (Figure 3c,f).

### 3.5 ∣ Diffusion Through Hydrated Collagen Matrices

**FIGURE 4 ∣:**
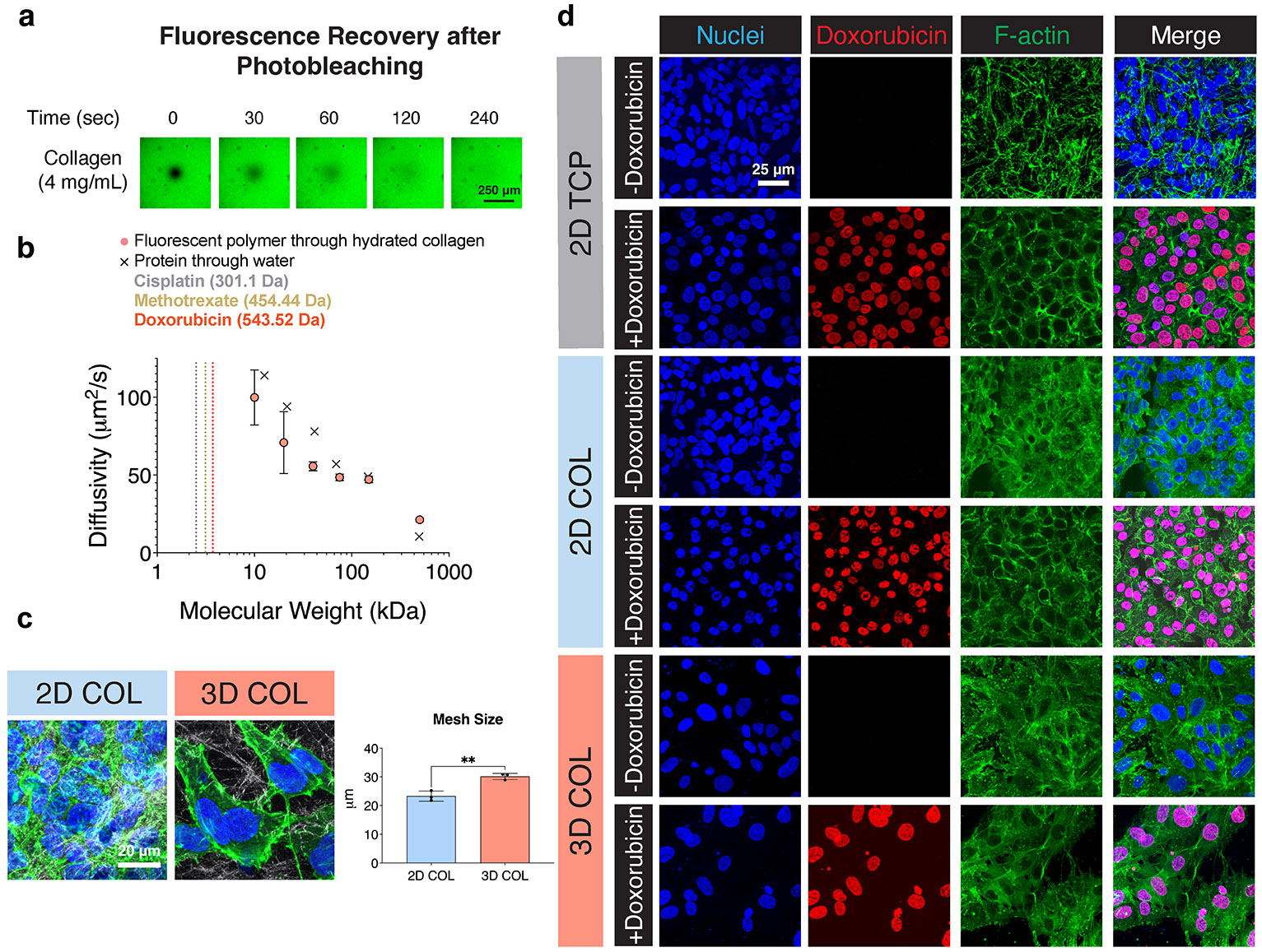
Analysis of diffusivity, mesh size at day 7 of culture, and drug penetration in COL matrices. (a) Representative fluorescence recovery after photobleaching images showing fluorescent polymer diffusion through the COL matrix immediately after regional photobleaching (0 s) and following 240 s of signal recovery. Scale bar = 250 μm. (b) Diffusivity values for selected molecular weight polymers through the COL matrix, with over-layed diffusivity values of different sized proteins diffusing in water for comparison (*n* = 4 independent technical replicates, mean ± SD). The molecular weight sizes of the three chemotherapy drugs are indicated with vertical lines. (c) Representative SHG images of cell-laden 2D COL and 3D COL hydrogels after 7 days of culture demonstrated cell spreading throughout the matrix in both COL conditions. COL fibers imaged with SHG signal (white), nuclei stained with DAPI (blue), and F-Actin stained with phalloidin (green). Scale bar = 20 μm. Quantification of COL gel mesh size showed a decrease in mesh size in 2D COL compared to 3D COL. (*n* = 3 independent technical replicates, mean ± SD, unpaired two-tailed *t*-test, **p < 0.01) (d) Confocal microscopy of 143B OS cells after 24 h of incubation with 25 μM DOX. DOX (red) colocalizes with the DAPI nuclear stain (blue) and is largely excluded from the cytoplasm as indicated by the phalloidin F-Actin stain (green). Scale bar = 25 μm. Abbreviations: COL = collagen; SD = standard deviation; 2D = two-dimensional; 3D = three-dimensional; SHG = second harmonic generation; OS = osteosarcoma; DOX = doxorubicin.

In clinical presentations of solid tumors, restricted drug transport to the primary tumor is reported to prevent the efficacy of systemic chemotherapy. In our in vitro OS culture model, no vasculature is present; thus, drug transport occurs solely through passive diffusion. To assess whether diffusion-limited drug penetration may be contributing to the observed chemoresistance in the 3D COL condition, we quantitatively measured the diffusivity through our hydrated COL matrix. Fluorescence recovery after photobleaching is a technique to track the movement of fluorescent molecules of different sizes as they diffuse through a matrix over time (0–240 s) after laser-induced photobleaching of a focal region (Figure 4a). Computational fitting of the observed fluorescent recovery time is used to calculate the diffusivity parameter for six different molecular weight model molecules, fluorescent dextran polymers (10 to 500 kDa). Dextran is a water-soluble polymer that is commonly used in fluorescence after photobleaching evaluations to approximate the diffusion of various biomolecules through hydrogels and matrices.

As expected, as the molecular weight of the polymer increases, its diffusivity through the 3D COL matrix decreases, since larger objects diffuse more slowly (Figure 4b). With the technical limitations of our camera speed, the fastest diffusivity we could measure was ~100 μm^2^/s for the 10-kDa dextran. When comparing our quantitative data to the reported diffusivity values for different sized proteins moving through water [44], we find the values are all within a factor of two. These comparable diffusivity values suggest that diffusion through the hydrated 3D COL matrix is not significantly impeded by the presence of the COL fibers. COL mesh size, quantified after 7 days of culture in both the 2D COL and 3D COL culture conditions, remained unchanged from after cell seeding (Figure S6) and > 20 μm (Figure 4c), which is three orders of magnitude larger than our largest dextran polymer (diameter of ~30 nm for molecular weight of 500 kDa) [45]. For comparison, the molecular weights of the chemotherapy drugs MTX, DOX, and CIS (indicated by vertical lines in Figure 4b) are all well below the smallest polymer tested (10 kDa). Taken together, these data strongly suggest that these drugs would likely exhibit high diffusivity within the 3D matrix.

These results indicate that diffusion is unlikely to be a contributing cause for the differential chemotherapy response observed between 2D and 3D COL cultures. To further confirm drug penetration into cells in all three of our culture models, we evaluated nuclear localization of DOX after the 24-h drug treatment. DOX, an anthracycline chemotherapeutic, disrupts topoisomerase II DNA repair and intercalates into DNA, causing both single-strand and double-strand breaks [46]. Its intrinsic fluorescence at an excitation wavelength of 470 nm and emission wavelength of 560 nm allows for direct visualization of the drug following cellular fixation. We observed a 1:1 correlation between DOX and nuclear DAPI staining, indicating drug penetration into the cell nuclei within the 2D TCP, 2D COL, and 3D COL conditions (Figure 4d). This analysis confirms that diffusion is not a barrier to drug transport within the 3D COL matrix at a matrix density of 4 mg/mL.

### 3.6 ∣ Metronomic Treatment Regimen With Regorafenib

**FIGURE 5 ∣:**
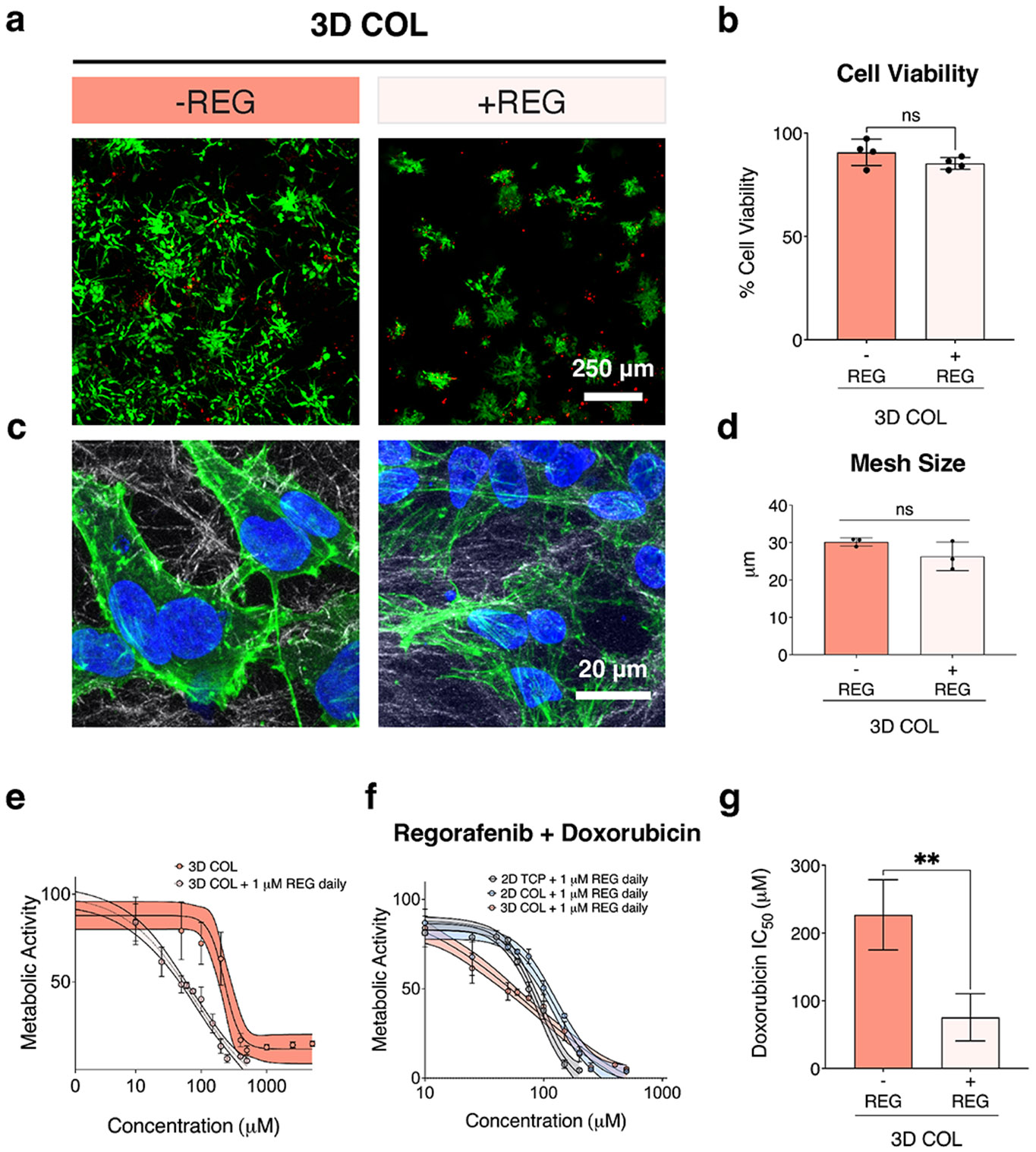
REG sensitizes OS cells cultured in 3D COL to DOX. (a, b) Cell viability remained high with no significant differences after 6 days of pre-treatment with REG in 3D COL culture. Scale bar = 250 μm. (*n* = 4 independent technical replicates, mean ± SD, ordinary one-way ANOVA with Tukey's post hoc analysis, no statistical differences). (c) Cell morphology was altered after REG pre-treatment as indicated by decreased cell spreading. COL fibers imaged with SHG signal (white), nuclei stained with DAPI (blue), and F-Actin stained with phalloidin (green). Scale bar = 20 μm. (d) Quantification of COL gel mesh size after REG pre-treatment showed no significant differences in local mesh size. (*n* = 3 independent technical replicates, mean ± SD, unpaired two-tailed *t*-test, **p < 0.01) (e) Dose response analysis after 6 days pre-treatment of REG and 24 h treatment of DOX in 3D COL culture showed an increase in chemosensitivity of cells pre-treated with REG (*n* = 4 independent technical replicates, mean ± SD, center solid line is nonlinear least squares regression of data, shaded region represents 95% confidence bands of the nonlinear fit) (f) REG pre-treated cells in 2D TCP, 2D COL, and 3D COL showed similar dose response curves after 24 h of treatment with DOX (*n* = 4 independent technical replicates, mean ± SD, center solid line is nonlinear least squares regression of data, shaded region represents 95% confidence bands of the nonlinear fit). (g) Pre-treatment with REG decreased the IC_50_ of OS cells in 3D COL, indicating increased chemosensitivity to DOX (*n* = 4 independent technical replicates, IC_50_ values calculated from nonlinear fit of dose–response curves, unpaired two-tailed *t*-test, **p < 0.01). Abbreviations: REG = regorafenib; OS = osteosarcoma; 3D = three-dimensional; COL = collagen; DOX = doxorubicin; SD = standard deviation; ANOVA = analysis of variance; SHG = second harmonic generation; 2D = two-dimensional; TCP = tissue culture plastic.

To simulate current treatment protocols for high-risk OS patients, we generated a clinically relevant metronomic chemotherapy regimen using regorafenib (REG), a tyrosine kinase inhibitor used off-label to treat relapsed and refractory OS patients with metastatic disease [47, 48]. In this approach, cells were treated daily with a low dose of REG (1 μM) for 6 days, starting at day 1 of culture, with the aim of sensitizing cells to DOX. OS cells treated with daily 1 μM REG maintained ~75% metabolic activity compared to no REG control, while daily treatment with 5 μM REG yielded a significant decrease in metabolic activity to ~5% in all culture systems (Figure S7). Despite the daily 1 μM REG treatment, cells in 3D COL remained viable after 6 days of treatment, though they exhibited reduced cell spreading within the ECM compared to untreated controls (Figure 5a-c). Pre-treatment with REG did not alter local COL mesh size as quantified by SHG imaging (Figure 5d). Cells cultured in 2D TCP and 2D COL exhibited no change in cell morphology or viability upon REG treatment (Figure S8a,b).

To determine whether REG could overcome ECM-induced chemoresistance, we measured the response to DOX after a 6-day REG treatment. We observed a left shift in the DOX dose–response curve in REG-pretreated 3D COL cultures compared to untreated 3D COL cultures (Figure 5e). Additionally, after daily pre-treatment with REG and 24-h treatment with DOX, the DOX dose response of cells in 2D TCP, 2D COL, and 3D COL cultures was approximately similar (Figure 5f). Taken together, the IC_50_ value in REG-pretreated 3D COL cultures was significantly lower than that for untreated 3D COL cultures (Figure 5g), demonstrating that REG can increase OS cell sensitivity to DOX in our 3D COL model.

### 3.7 ∣ Expression of ABC Transporters in 3D COL Culture

**FIGURE 6 ∣:**
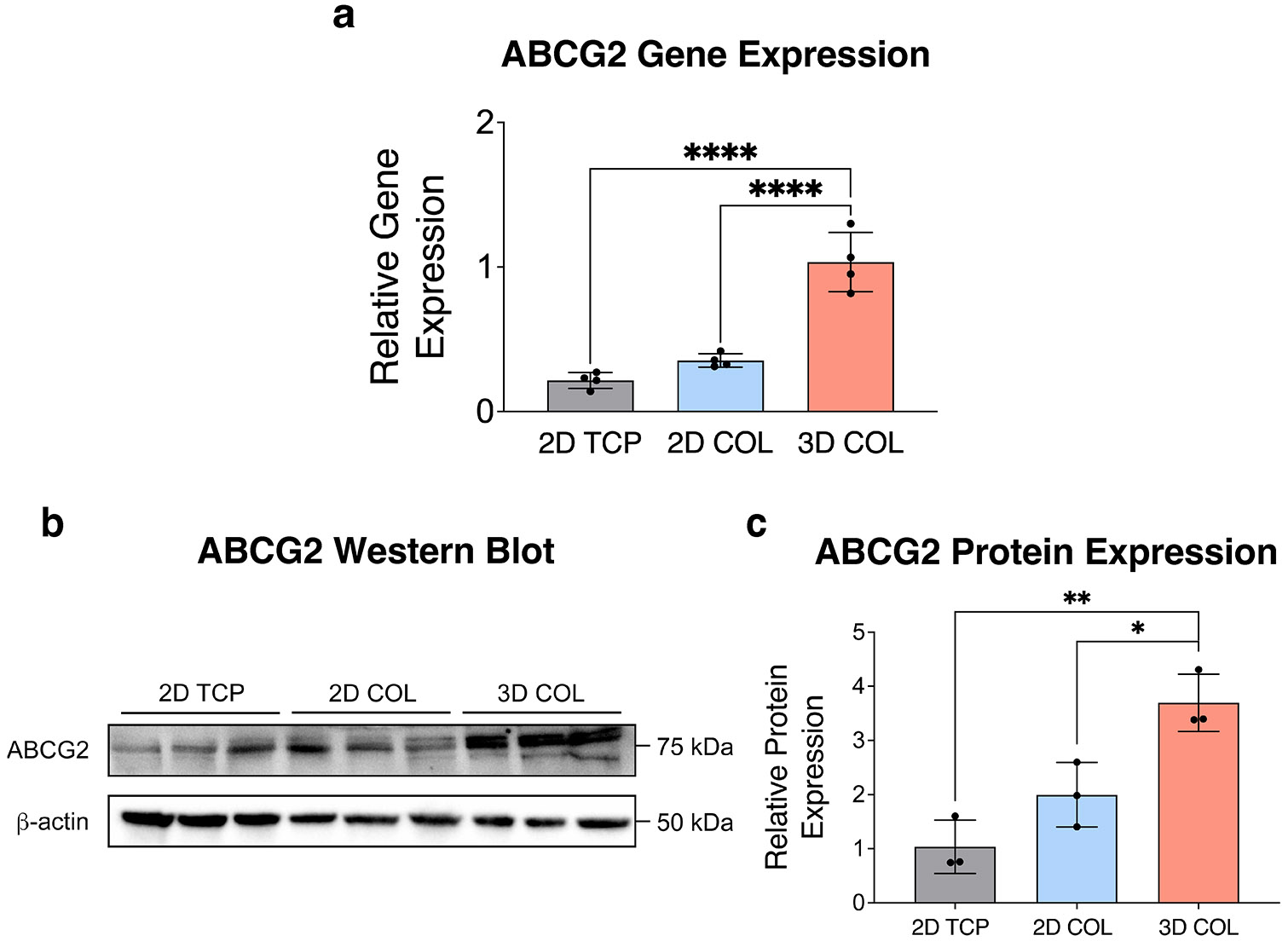
Elevated ABCG2 expression in OS cells cultured in 3D COL matrices. (a) Gene expression of ABCG2, a drug efflux pump, was significantly upregulated in 3D COL cultures compared to 2D TCP and 2D COL cultures (*n* = 4 independent technical replicates, mean ± SD, ordinary one-way ANOVA with Tukey's post hoc analysis, ****p < 0.0001). (b, c) ABCG2 protein expression was markedly higher in 3D COL culture compared to 2D TCP and 2D COL (*n* = 3 independent technical replicates, mean ± SD, ordinary one-way ANOVA with Tukey's post hoc analysis, *p < 0.05, **p < 0.01). Abbreviations: ABC = ATP-binding cassette; OS = osteosarcoma; 3D = three-dimensional; COL = collagen; TCP = tissue culture plastic; SD = standard deviation; ANOVA = analysis of variance.

We next investigated whether ABC transporters, drug efflux pumps correlated with OS chemoresistance, were upregulated in 3D culture. Others have demonstrated that cell–matrix interactions can modulate ABC transporter expression, which is known to correlate with tumor chemosensitivity [31, 32]. Further, REG allosterically inhibits the ATP-binding sites of ABCB1 and ABCG2, functioning as an efflux pump inhibitor. First, we assessed gene expression of ABC transporters on day 7 of culture, before chemotherapy treatment, as a potential mechanism of chemoresistance in 3D COL culture. We observed increased expression of *ABCG2* (Figure 6a) and *ABCC1* (Figure S9a) in 3D COL compared to 2D TCP and 2D COL, while there was no significant difference in gene expression of *ABCB1* (Figure S9b). Since both MTX and DOX are substrates for ABCG2, we focused on this transporter as a potential mechanism behind the observed chemoresistance in 3D COL. [49] Western blot analysis confirmed elevated protein levels of ABCG2 in 3D COL cultures relative to 2D COL and 2D TCP (Figure 6b,c). These findings highlight the potential role of ABCG2 in mediating chemoresistance in 3D COL culture and suggest a mechanism behind the restoration of chemosensitivity with REG pre-treatment.

## 4 ∣ Discussion

Consequences of cell–matrix interactions between OS cells and COL, the primary matrix component of malignant bone, remain undefined. Characterizing changes in cell phenotype in response to the ECM could lead to drug targets and define new approaches to OS treatment regimens. By generating a tractable model of OS through a 3D culture platform, we were able to demonstrate that 3D culture within COL hydrogels led to significant alterations in cell phenotype. From a biomaterials perspective, the COL hydrogels used in this study were fabricated at a matrix density of 4 mg/mL and exhibited a stiffness of ~2.4 kPa. This modulus mimics native bone marrow at physiological temperature, which is reported to range from 0.1 to 10.9 kPa [50]. In contrast, the elastic modulus of TCP ranges from 1 to 10 GPa [51]. SHG microscopy confirmed a disorganized fibril/fiber structure similar to the randomly organized collagen fibers of osteoid in native OS tumors, facilitating integrin binding to the COL fibers [52].

The presence of COL induced upregulation of *AKT* gene expression and activated, phosphorylated AKT protein levels in both 2D and 3D culture conditions, suggesting the matrix could effectively induce integrin receptor engagement and downstream signaling in both 2D and 3D COL cultures. In contrast, we observed significant differences between 2D and 3D COL culture through examination of cell morphology and ALP activity, a biomarker of OS, which is upregulated at patient diagnosis (Figure 2a) [40]. These results suggest that dimensionality can directly influence cell phenotype, aligning with the findings by Tornín et al., who reported increased ALP activity in MG-63 OS cells cultured within a 3D COL-hydroxyapatite scaffold over a 9-day in vitro culture period [53]. Clinically, elevated ALP levels at diagnosis are associated with poor prognosis marked by reduced overall and disease-free survival [54]. Dose–response curves to MTX, DOX, and CIS, the three agents used in front-line therapy for OS, suggested that the 143B OS cells are less chemosensitive when cultured in 3D environments compared to 2D culture models. This finding is consistent with other studies showing that 3D OS cultures within biomimetic bone matrices composed of both natural and synthetic biopolymers or scaffold-free environments show decreased chemosensitivity compared to 2D TCP cultures [22-24, 27, 55-60]. Each of these studies confirms the need to study OS cells within 3D systems that are more indicative of the in vivo microenvironment due to differential gene and protein expression as well as response to chemotherapy. Taken together, our data suggest that although the OS cells can engage in downstream biochemical signaling in response to COL in both 2D and 3D models (Figure 2a-c), the dimensionality of the culture model (i.e., 2D vs. 3D) results in different cell phenotypes in terms of chemotherapy response (Figure 3). Our analysis of diffusion both through fluorescence recovery after photobleaching imaging and quantification (Figure 4a,b), local mesh size after 7 days of culture (Figure 4c), as well as nuclear incorporation of DOX (Figure 4d) confirmed that diffusion and drug penetration through the matrix was not a limiting factor and likely not the mechanism behind the decreased chemosensitivity response of cells cultured within 3D COL environments.

To overcome cell-matrix induced drug resistance, we generated a clinically relevant metronomic treatment regimen employing a tyrosine kinase inhibitor used in relapsed and refractory OS patients. Several tyrosine kinase inhibitors including REG, cabozantinib, and sorafenib have been evaluated in recent phase II clinical trials for refractory and relapsed OS patients [47, 61]. The commonly reported mechanism of action for TKIs is the inhibition of receptor tyrosine kinases, which have been implicated in various cancers including OS [62]. TKIs interfere with these pathways in a nonspecific manner, affecting multiple receptor tyrosine kinases involved in OS growth, such as those regulating tumor angiogenesis (vascular endothelial growth factor receptor •1, •2, and •3) and cellular recruitment to the TME (platelet derived growth factor and fibroblast growth factor receptor) [63].

Our proposed regimen involved daily pre-treatment with 1 μM of REG (days 1–6) prior to DOX treatment on day 7 of culture. Indeed, we accordingly observed a left shift in the dose–response curve (Figure 5e) with REG pre-treatment in 3D COL, with a significant decrease in the IC_50_ value (Figure 5g). We explored the expression of ABC transporters as a potential mechanism behind reduced chemosensitivity of OS cells cultured in 3D COL. We observed increased gene expression of *ABCG2* and *ABCC1* (Figures 6a and S9a) and confirmed protein expression of ABCG2 (Figure 6c) in OS cells cultured in 3D COL. These data correlate with the increases in *AKT* gene expression and activated AKT protein levels, as activation of the PI3K/AKT pathway modulates multidrug resistance through increased expression of ABC transporters, specifically ABCG2 and ABCC1 [64]. Further, others have demonstrated an increase in ABC transporter expression of OS cells cultured within 3D matrices [23].

Tyrosine kinase inhibitors have been found to inhibit ABC transporters by blocking the ATP-binding site, which reduces chemotherapy efflux by preventing downstream phosphorylation [65]. Notably, REG has drug–drug interactions with substrates of ABCG2/BCRP due to its inhibitory effect on ABCB1 and ABCG2 [66, 67]. Recent work by Zhang et al. demonstrated that REG can inhibit ABCG2, thereby increasing chemotherapy accumulation within OS cells both in vitro and in vivo [68]. The authors found that REG did not lead to changes in gene or protein expression of ABCG2 but rather stimulated ABCG2 ATPase activity, thereby preventing the function of the efflux pump. While the results from this study may involve multiple mechanisms beyond ABC transporter inhibition, these data suggest a promising clinical approach for combinatorial treatment using tyrosine kinase inhibitors with conventional chemotherapy at lower doses to potentially achieve similar levels of OS cell death with less severe off-target effects.

Concurrent or sequential use of tyrosine kinase inhibitors with cytotoxic chemotherapy has demonstrated efficacy in multiple pediatric cancer protocols through the Children's Oncology Group. These studies have investigated various TKIs such as sorafenib and pazopanib and have spanned multiple cancers, including pediatric hepatocellular carcinoma (AHEP1531) and non-rhabdomyosarcoma (ARST1321) [69, 70]. REG has clinical activity in patients with relapsed, refractory, or progressive metastatic OS, yielding a positive effect on delaying disease progression after failure in response to MAP therapy [47, 48]. However, none of these trials have achieved complete remission and cured patients of their disease. Our data suggest that daily administration of a low dose of REG may sensitize OS cells prior to standard chemotherapy. Further, our results demonstrating a decrease in the IC_50_ upon REG pre-treatment indicate a lower dose of chemotherapy could be effective, potentially leading to decreased toxic acute and long-term side effects from standard chemotherapy.

This work is limited by the use of a single OS cell line and will need to be expanded to other OS cell lines, patient-derived cell lines, and preclinical patient-derived xenograft models. Additionally, future studies can modify our model to study other variables of the TME such as COL matrix density, stiffness, and orientation, as well as other ECM components such as fibronectin and laminin. To further enhance the model, other key cell types within the OS TME including macrophages, bone marrow stromal cells, and endothelial cells could be added. Constructs can also be evaluated within hypoxic environments, a known driver of chemoresistant disease. Finally, future long-term studies could mimic the full, multi-week MAP chemotherapy cycle, which involves administering DOX and CIS on days 1 and 2 sequentially, followed by MTX on weeks 4 and 5. Future work correlating ABC transporter expression in metastatic, relapsed, or refractory status could guide the concurrent or sequential use of TKIs such as REG to sensitize malignant cells to frontline chemotherapy. A combinatorial approach with cabozantinib, a multi tyrosine kinase inhibitor, and MAP chemotherapy is now underway in the current Phase 2/3 study from the Children's Oncology Group to treat newly diagnosed OS. Our work underlines the potential benefit of this combinatorial tyrosine kinase inhibitor plus chemotherapy approach to enhance treatment outcomes of high-risk OS patients.

## 5 ∣ Conclusions

Our study reveals that culturing 143B OS cells within a 3D COL matrix induces distinct cellular responses leading to alterations in cell morphology, increased ALP activity, and decreased chemosensitivity compared to 2D TCP and 2D COL cultures. Interestingly, we observed that both 2D COL and 3D COL cultures increased PI3K/AKT pathway gene expression. Taken together, these results suggest that dimensionality (rather than the simple presence of COL) is required to observe these phenotypic differences in cell morphology and chemosensitivity. Importantly, we found that diffusion through the 3D COL matrix was not a limiting factor to drug penetration through the matrix. As a reproducible, affordable in vitro assay, our 3D COL matrix model is well poised for future studies seeking to identify drug targets and their mechanisms of action. Pre-treatment with REG, a tyrosine kinase inhibitor used to treat relapsed and refractory OS patients, lowered the IC_50_ value for 3D COL cultures, indicating improved drug sensitivity. OS cells cultured within this 3D microenvironment exhibited elevation in ABCG2 gene and protein expression. Our findings suggest that combining REG with lower doses of chemotherapy could achieve similar efficacy as higher doses of chemotherapy alone. This strategy offers a promising approach for overcoming ECM-induced reduction in chemosensitivity of OS cells, potentially leading to improved patient outcomes in clinical settings.

## Supplementary Material

Supplemental Figures and Table

Additional supporting information can be found online in the Supporting Information section. **Data S1:** Supporting Information.

## Data Availability

The data that support the findings of this study are openly available in Dryad at https://doi.org/10.5061/dryad.w3r228128, reference number JBMR-A-25-0496.
