# Supplementary material for: Targeting Cell-Matrix Induced Chemoresistance With Regorafenib in a 3D Model of Osteosarcoma
Source: J Biomed Mater Res A. Author manuscript; Available in PMC 2025 Nov 13. (PMC12614213; doi:10.1002/jbm.a.37985)
Supplement: Supplemental Figures and Table [file NIHMS2121562-supplement-Supplemental_Figures_and_Table.docx]

**Targeting Cell-Matrix Induced Chemoresistance with Regorafenib in a 3D Model of Osteosarcoma**

Rameshwar R. Rao^1,2^*, Michelle S. Huang^3^, Daiyao Zhang^3^, Carla Huerta-López^4^, Christopher Long^4^, Giselle Aviles Rodriguez^4^, Esther A.T. Mozipo,^5^ Sriya Sagi^1^, Sarah C. Heilshorn^4^*

R.R. Rao ([ram.rao@seattlechildrens.org](mailto:ram.rao@seattlechildrens.org))

S. Sagi ([sriya.sagi@seattlechildrens.org](mailto:sriya.sagi@seattlechildrens.org))

1. Ben Towne Center for Childhood Cancer and Blood Disorders Research, Seattle Children’s Research Institute, Seattle, WA, 98101, USA

2. Division of Pediatric Hematology, Oncology, Bone Marrow Transplant, and Cellular Therapies, Department of Pediatrics, University of Washington School of Medicine, Seattle, WA, 98105, USA

M.S. Huang ([michuang@stanford.edu](mailto:michuang@stanford.edu))

D. Zhang ([daiyao@stanford.edu](mailto:daiyao@stanford.edu))

3. Department of Chemical Engineering, Stanford University, 466 Lomita Mall, Stanford, CA, 94305, USA

C. Huerta-López ([carlahl@stanford.edu](mailto:carlahl@stanford.edu))

C. Long ([cmlong@stanford.edu](mailto:cmlong@stanford.edu))

G. Aviles Rodriguez ([giselleaviles018@gmail.com](mailto:giselleaviles018@gmail.com))

S.C. Heilshorn ([heilshorn@stanford.edu](mailto:heilshorn@stanford.edu))

4. Department of Materials Science and Engineering, Stanford University, 466 Lomita Mall, Stanford, CA 94305, USA

E. Mozipo ([emozipo@stanford.edu](mailto:emozipo@stanford.edu))

5. Department of Bioengineering, Stanford University, 443 Via Ortega, Stanford, CA, 94305, USA

* Co-corresponding authors

**Supplementary Figures and Tables**


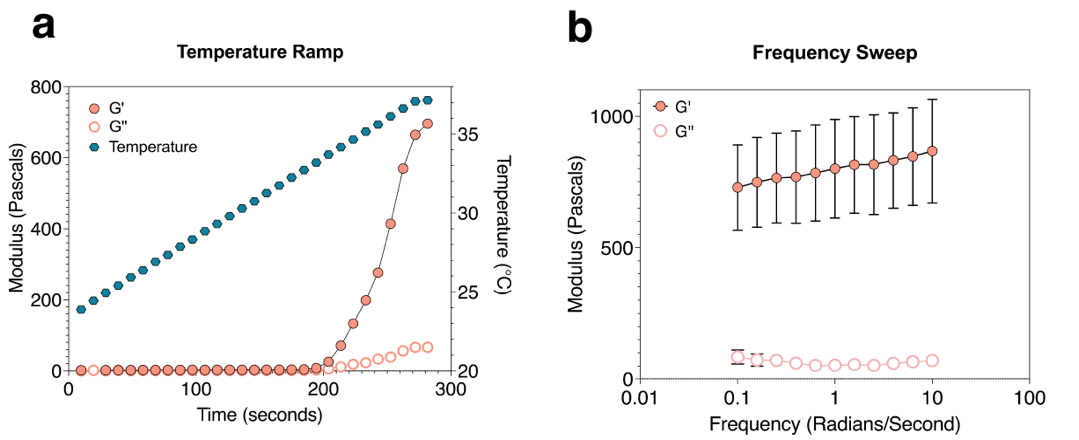


**Figure S1. Characterization of COL matrix mechanical properties.** (**a**) Rheological analysis showing the temperature-dependent gelation of COL, with hydrogel formation occurring within 3 minutes, as indicated by the crossover point of the storage modulus (G’) and loss modulus (G’’). (**b**) Frequency sweep of the fully formed COL hydrogel, demonstrating an average storage modulus of approximately 800 Pa at 1 radian/second (n = 4 independent hydrogel replicates, mean +/- SD). Abbreviations: COL = collagen; Pa = pascals; SD = standard deviation.

**
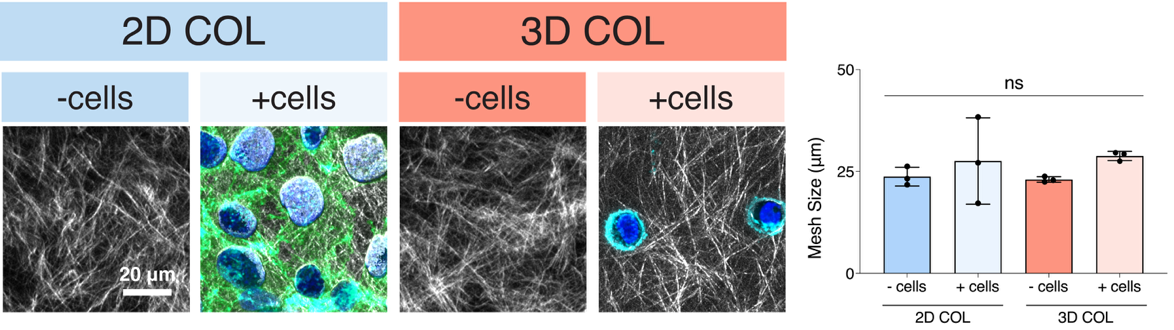
**

**Figure S2. Second harmonic generation (SHG) imaging of acellular and cell-laden 2D COL and 3D COL hydrogels.** Representative SHG images of acellular and cell-laden 2D COL and 3D COL hydrogels fixed 30 minutes after cell-seeding and immediately after gelation, respectively. COL fibers imaged with SHG signal (white), nuclei stained with DAPI (blue) and F-actin stained with phalloidin (green). Quantification of COL gel mesh size revealed no significant differences between 2D COL and 3D COL matrices with and without cells (n = 3 independent technical replicates, mean +/- SD, ordinary one-way ANOVA with Tukey’s post hoc analysis, ns = not significant). Abbreviations: SHG = second harmonic generation; 2D = two-dimensional; COL = collagen; 3D = three-dimensional; SD = standard deviation; ANOVA = analysis of variance.


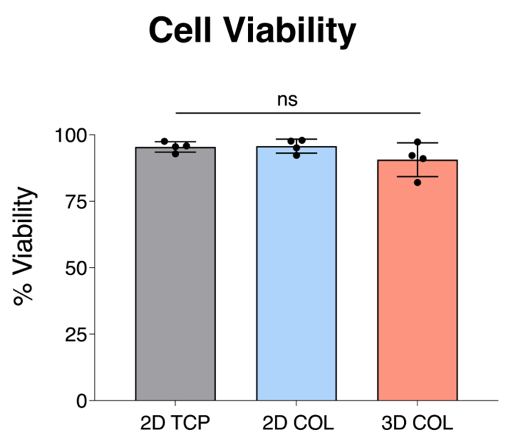


**Figure S3.** **Cell viability on day 7 of culture**. Cell viability of OS cells cultured in 2D TCP, 2D COL, and 3D COL showed no significant differences on day 7 (n = 4 independent technical replicates, mean +/- SD, ordinary one-way ANOVA with Tukey’s post hoc analysis, ns = no statistical differences). Abbreviations: OS = osteosarcoma; 2D = two-dimensional; TCP = tissue culture plastic; COL = collagen; 3D = three-dimensional; SD = standard deviation; ANOVA = analysis of variance.


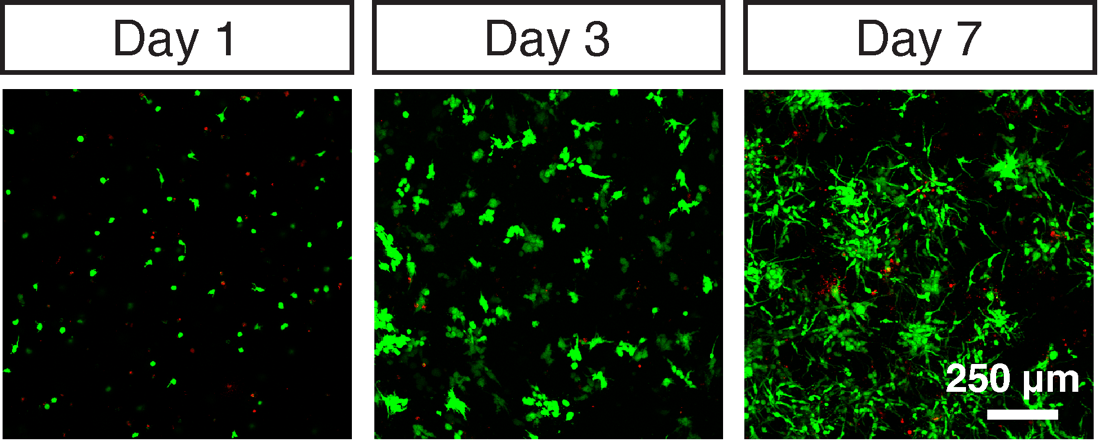


**Figure S4.** **Viability of OS cells on days 1, 3, and 7 in 3D COL.** Live/Dead staining of OS cells in 3D COL at days 1, 3, and 7 of culture, showing high cell viability and homogenous distribution of cells through the matrix (green – live cells, red – dead cells). Multicellular units formed at day 3 of culture and cells elongated at day 7 of culture. Scale bar = 250 μm. Abbreviations: OS = osteosarcoma; 3D = three-dimensional; COL = collagen.


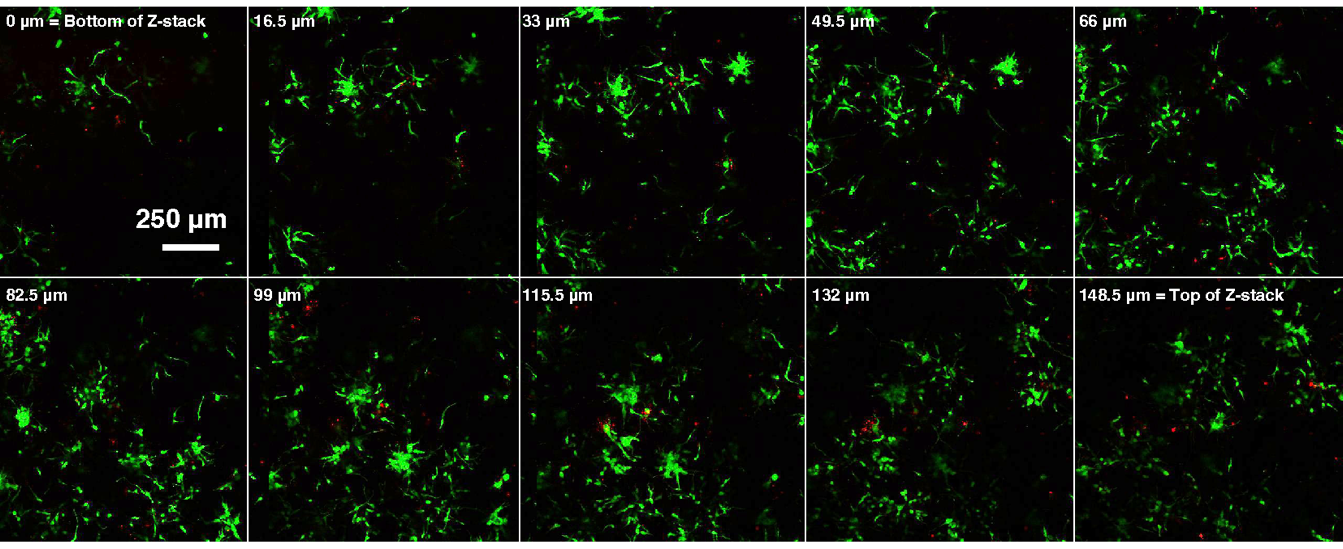


**Figure S5. Montage of z-stack of cells within 3D COL.** Z-stack of ten images through a cell-laden 3D COL as depicted in Figure 1d. Scale bar = 250 μm. Abbreviations: 3D = three-dimensional; COL = collagen.

**
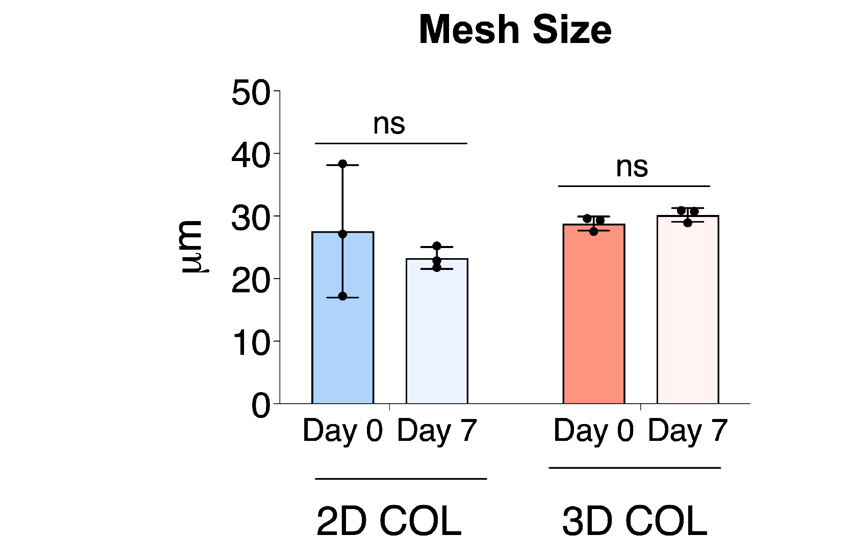
**

**Figure S6.** **COL mesh size throughout the 7-day culture period.** Quantification of COL gel mesh size from SHG images revealed no significant differences between day 0 and day 7 of culture in either 2D COL or 3D COL matrices with cells (n = 3 independent technical replicates, mean +/- SD, unpaired two-tailed t-test, ns = not significant). Abbreviations: COL = collagen; SHG = second harmonic generation; 2D = two-dimensional; 3D = three-dimensional; SD = standard deviation; ANOVA = analysis of variance.

**
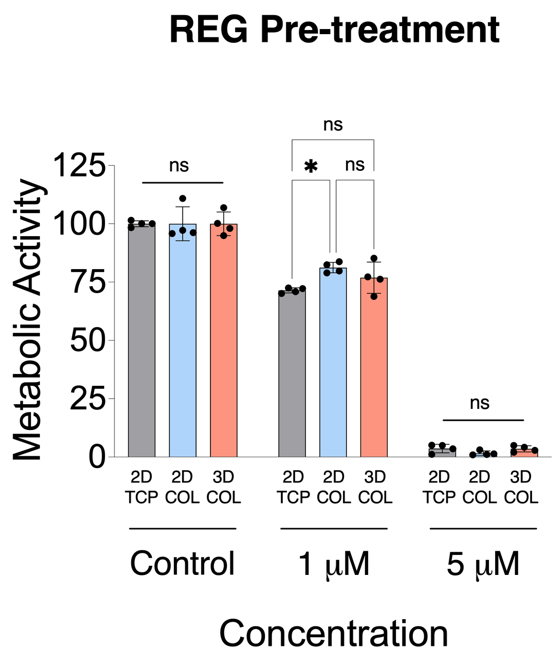
**

**Figure S7.** **Metabolic activity on day 7 after REG pre-treatment.** Cell metabolic activity of OS cultured in 2D TCP, 2D COL, and 3D COL after six days of pre-treatment with either no REG (control), 1 μM or 5 μM of REG. Values are normalized to control. Cells maintained ~75% metabolic activity in all three culture conditions after pre-treatment with 1 μM REG but demonstrated a significant decrease in metabolic activity (~5%) in all culture conditions with 5 μM REG pre-treatment. (n = 4 independent technical replicates, mean +/- SD, ordinary one-way ANOVA with Tukey’s post hoc analysis, ns = no statistical differences, * p < 0.05). Abbreviations: REG = regorafenib; OS = osteosarcoma; 2D = two-dimensional; TCP = tissue culture plastic; COL = collagen; 3D = three-dimensional; SD = standard deviation; ANOVA = analysis of variance.


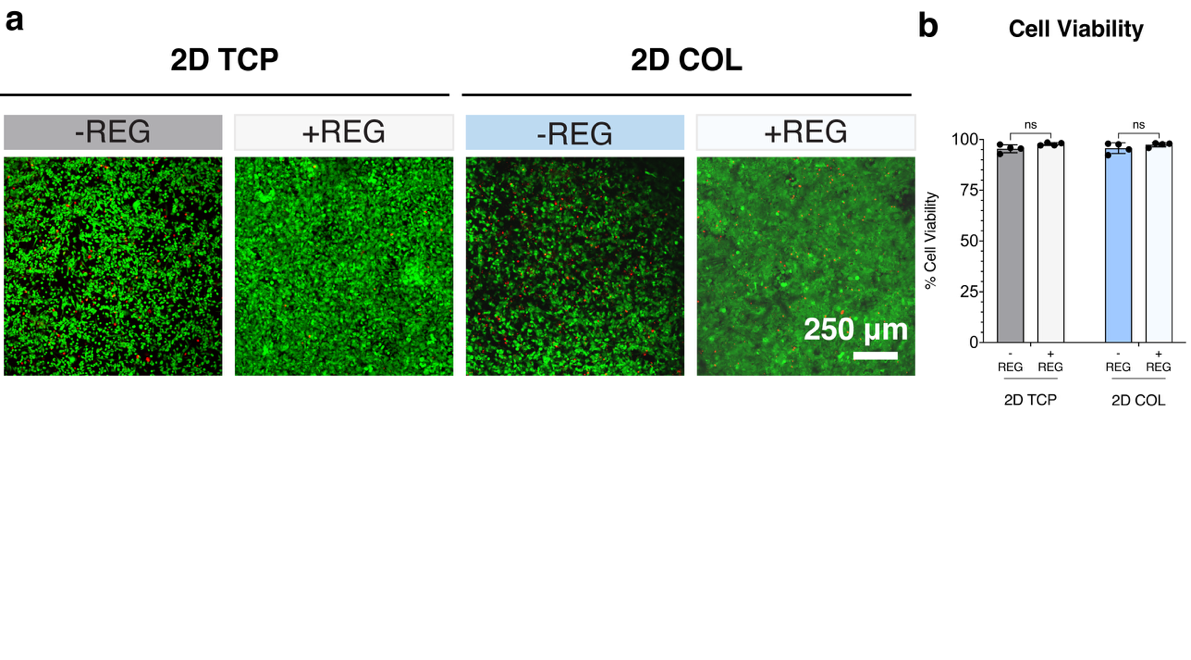


**Figure S8.** **Impact of REG pre-treatment on OS cells cultured on 2D TCP and 2D COL.** (**a**) Live/Dead staining of OS cells cultured in 2D TCP and 2D COL with and without 6 days of REG pre-treatment. (**b**) Cell viability remained consistently high across both conditions, with no significant differences (n = 4 independent technical replicates, mean +/- SD, mixed effects two-way ANOVA with Tukey’s post hoc analysis, no statistical differences). Abbreviations: REG = regorafenib; OS = osteosarcoma; 2D = two-dimensional; TCP = tissue culture plastic; COL = collagen; SD = standard deviation; ANOVA = analysis of variance.


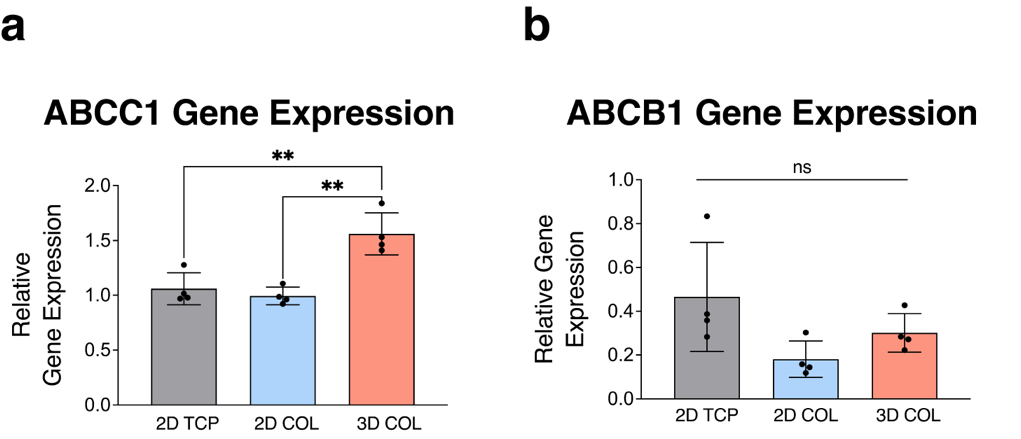


**Figure S9.** **ABC transporter gene expression**. (**a**) Gene expression of ABCC1 was significantly increased in 3D COL compared to 2D TCP and 2D COL (n = 4 independent technical replicates, mean +/- SD, ordinary one-way ANOVA with Tukey’s post hoc analysis, ** p < 0.01). (**b**) Gene expression of ABCB1 was not significantly different in 2D TCP, 2D COL, and 3D COL conditions (n = 4 independent technical replicates, mean +/- SD, ordinary one-way ANOVA with Tukey’s post hoc analysis, no statistical differences). Abbreviations: ABC = ATP-binding cassette; 3D = three-dimensional; COL = collagen; 2D = two-dimensional; TCP = tissue culture plastic; SD = standard deviation; ANOVA = analysis of variance.

**Table S1.** Human primers used for qRT-PCR experiments.

| **Gene** | **Forward Primer (5’ to 3’)** | **Reverse Primer (5’ to 3’)** |
| --- | --- | --- |
| AKT | AGCGACGTGGCTATTGTGAAG | GCCATCATTCTTGAGGAGGAAGT |
| ABCB1 | TTGCTGCTTACATTCAGGTTTCA | AGCCTATCTCCTGTCGCATTA |
| ABCC1 | CTCTATCTCTCCCGACATGACC | AGCAGACGATCCACAGCAAAA |
| ABCG2 | GGTGGAGGCAAATCTTCGTTA | GAGTGCCCATCACAACATCA |
| GAPDH | CATGAGAAGTATGACAACAGCCT | AGTCCTTCCACGATACCAAAGT |

**Table S2.** Antibodies used for imaging and Western blot experiments. Abbreviations: ABC = ATP-binding cassette; WB = western blot; AKT = protein kinase B; HRP = horseradish peroxidase; ICC = immunocytochemistry

| **Target** | **Host Species** | **Supplier** | **Catalog Number** | **Dilution** |
| --- | --- | --- | --- | --- |
| *Primary Antibodies* | | | | |
| ABCG2 | Rabbit | Cell Signaling Technology | 42078S | WB: 1:1000 |
| p-AKT | Rabbit | Cell Signaling Technology | 4060S | WB: 1:1000 |
| AKT | Rabbit | Cell Signaling Technology | 9272S | WB: 1:1000 |
| β-Actin | Rabbit | Cell Signaling Technology | 4970S | WB: 1:1000 |
| *Secondary Antibodies* | | | | |
| HRP-anti-rabbit | Donkey | Jackson ImmunoResearch | 711-035-152 | WB: 1:10,000 |
| *Other* | | | | |
| DAPI [5 mg/mL stock in water] |  | Millipore Sigma | D9542 | ICC: 1:1,000 |
| Alexa Fluor Plus 647 Phalloidin |  | Invitrogen | A30107 | ICC: 1:400 |
